# Mediating Effect of Illness Perception on the Relationship Between Perceived Family Function and Sleep Quality Among Patients With Atrial Fibrillation

**DOI:** 10.1002/nop2.70085

**Published:** 2024-11-22

**Authors:** Xiaoqing Lu, Zifen An, Yuying Xu, Xi Zhang, Pei Fang, Yaping Lu, Zhongxiang Cai, Liping Yu

**Affiliations:** ^1^ Cardiovascular Department Renmin Hospital of Wuhan University Wuhan Hubei Province China; ^2^ Wuhan University School of Nursing Center for Nurturing Care Research Wuhan University Wuhan Hubei Province China; ^3^ Nursing Department Zhongnan Hospital of Wuhan University Wuhan Hubei Province China; ^4^ Medical Center for Gastrointestinal Surgery Weifang People's Hospital Weifang Shandong China; ^5^ Teaching Department Renmin Hospital of Wuhan University Wuhan Hubei Province China; ^6^ Nursing Department Renmin Hospital of Wuhan University Wuhan Hubei Province China

**Keywords:** atrial fibrillation, family function, illness perception, sleep quality

## Abstract

**Aims:**

To evaluate the sleep quality of patients with atrial fibrillation and its influencing factors, and explore whether illness perception mediates the relationship between family function and sleep quality.

**Design:**

Cross‐sectional survey conducted from November 2020 to November 2021.

**Methods:**

A total of 191 participants validly completed the Pittsburgh Sleep Quality Index, the Family APGAR Index and the Brief Illness Perception Questionnaire. Data were analysed using descriptive statistics, independent samples *t*‐tests, one‐way ANOVA, Pearson correlation analysis and multiple linear regression analysis. Bootstrapping was used to detect the mediating role of illness perception.

**Results:**

Patients with atrial fibrillation reported poor sleep quality, good family function and a moderate level of illness perception. The better the family function, the lower the level of illness perception and the better sleep quality in patients with atrial fibrillation. Patients with commercial medical insurance had lower levels of sleep quality relative to self‐financed patients. EHRA III and EHRA IV patients had worse sleep quality than EHRA II patients. Illness perception played a significant mediating role in the relationship between family function and sleep quality.

**Conclusions:**

Patients with atrial fibrillation have poorer sleep quality, and the type of medical insurance and EHRA score are independent indicators related to their sleep quality. Future health education and interventions need to focus on strengthening and improving the emotional support of family members in order to improve family function and reduce illness perception, thereby improving sleep quality of patients with atrial fibrillation.

**Impact:**

This study provides further evidence that nurses need to enhance their awareness and provide ongoing education to better identify patients with AF who have family dysfunction and perceived high levels of illness threat perceptions, as these factors negatively impact sleep quality.

**Reporting Method:**

This study was reported in strict compliance with the strengthening the reporting of observational studies in epidemiology (STROBE) guideline.

**Patient or Public Contribution:**

No patient or public contribution.

## 1 Introduction

Atrial fibrillation (AF), the most prevalent persistent cardiac arrhythmia, with a worldwide estimate of up to 37,574 million cases in 2017, its incidence and prevalence are increasing worldwide, which is projected to increase by 63% and 66% by 2050 (Lippi, Sanchis‐Gomar, and Cervellin 2021). According to statistics, the crude incidence rate and mortality rate of AF in China in 2019 were 81.914/100,000 and 3.638/100,000, respectively, presenting a time‐varying trend of younger onset and higher mortality rate in the elderly (Zhou et al. 2022). A systematic review and meta‐analysis showed that AF can lead to an increased risk of a range of different outcomes, including increased risk of morbidity (61%–96% increase in cardiovascular disease and 64% higher risk of chronic kidney disease) and a 46% increase in risk of all‐cause mortality (Odutayo et al. 2016). As a result of this significant impact on public health, the increasing prevalence of AF worldwide has imposed a growing disability burden and economic burden (Chung et al. 2020; Morin et al. 2016).

Among the various aspects of AF management, sleep quality has emerged as a pivotal factor influencing disease progression and patient well‐being. Sleep is an individual's fundamental, active state with dynamic physiological processes (Kwon et al. 2018), and its quality is the embodiment of individual self‐satisfaction with all aspects of sleep experience (Nelson, Davis, and Corbett 2022), which has been proven to be related to heart disease and recovery (Deschênes et al. 2019). Previous studies have reported that poor sleep quality is common in patients with AF (Risom et al. 2018; Szymanski et al. 2014; Wood, Higgins, and Barnes 2023). Christensen et al. (2018) found that sleep quality deprivation itself may be a key risk factor for AF due to the effect of sleep quality on autonomic nervous tension. Therefore, in the process of clinical intervention, identifying and improving sleep quality in patients with AF are of great significance in reducing the risk of AF and its associated complications.

## 2 Background

In clinical practice, nurses play a vital role in managing patients with AF and addressing their multifaceted needs (van den Dries et al. 2020). However, the current nursing practices for patients with AF often focus on pharmacological and procedural interventions, with less emphasis on the psychological and social factors that significantly impact sleep quality. More psychological load (anxiety, depression, etc.) and higher AF symptom severity are often associated with poorer sleep quality in AF patients (Risom et al. 2018), which may stem from the fact that an individual's emotional appraisal and perceptual understanding of the disease can influence their disease experience.

Illness perception has been defined as a dimensional structure that explores an individual's beliefs and representations of illness, which can guide coping to manage the entire illness experience (Leventhal, Meyer, and Nerenz 1980). A study of illness perception in patients with recurrent symptomatic AF found that illness perception had the strongest impact on psychological distress, contributing more than coping strategies and symptom frequency to psychological distress (McCabe and Barnason 2012). The results of a recent meta‐analysis also showed that cognitive‐behavioural therapy is very effective in improving sleep patterns in patients with insomnia (van Straten et al. 2018). However, studies describing the impact of illness perception on sleep quality in patients with AF are scarce, particularly from a nursing perspective.

Social support is an externalising factor that has been recognised as helping to mitigate the harmful effects of negative events and stress on individuals (Gable and Bedrov 2022). Ding et al. (2023) found that the level of social support perceived by patients with AF affects their attitudes towards the disease and that having better social support contributes to positive illness perception. Family is the most fundamental unit of life in human society. The degree of family function in adversity is the embodiment of communication, coping with and adapting to family stress and intimacy among family members (Zhang 2018). Although supportive social relationships from the family have been proven to be closely related to individual sleep quality (Ailshire and Burgard 2012; Mousavi et al. 2022; Stafford et al. 2017), it remains unclear whether the level of family function in patients with AF is related to sleep quality. Given the importance of family support in buffering stress and promoting resilience, understanding the interplay among family function, illness perception and sleep quality in patients with AF is crucial for developing targeted nursing interventions.

To better understand the intricate relationship among family function, illness perception and sleep quality in patients with AF, the ABC‐X model, an influential theoretical framework for analysing family stress and coping, offers valuable insights. Originating from Hill (1949) and further elaborated by Jurczak and Hill (1950), the ABC‐X model encapsulates a holistic view of family resilience and adaptation in the face of adversity. The ABC‐X model posits that stress within families arises from an interplay of three contextual factors: (A) the events that cause stress in the individual or family system, and (B) the resources or strength of the family to deal with the stress. These stressors lead to direct family stress reactions (C), manifesting as altered emotional states, communication patterns and problem‐solving behaviours. Finally, the model accounts for coping and adaptation mechanisms (X) employed by the family system to buffer the adverse effects of stress (Rosino 2016). When families experience stressful events, the meaning given by families to stressful events is critical in determining whether the severity of a family's stress causes a family crisis. From these perspectives, we speculate that illness perception may play an intermediary role between family function and sleep quality in patients with AF.

Our study aims to address this gap by evaluating the sleep quality of patients with AF and exploring the mediating effect of illness perception on the relationship between perceived family function and sleep quality. By doing so, we hope to provide new insights and strategies for nursing practice, enabling nurses to better support patients with AF in improving their sleep quality and overall well‐being.

## 3 Methods

### 3.1 Aims and Hypotheses

**FIGURE 1:**
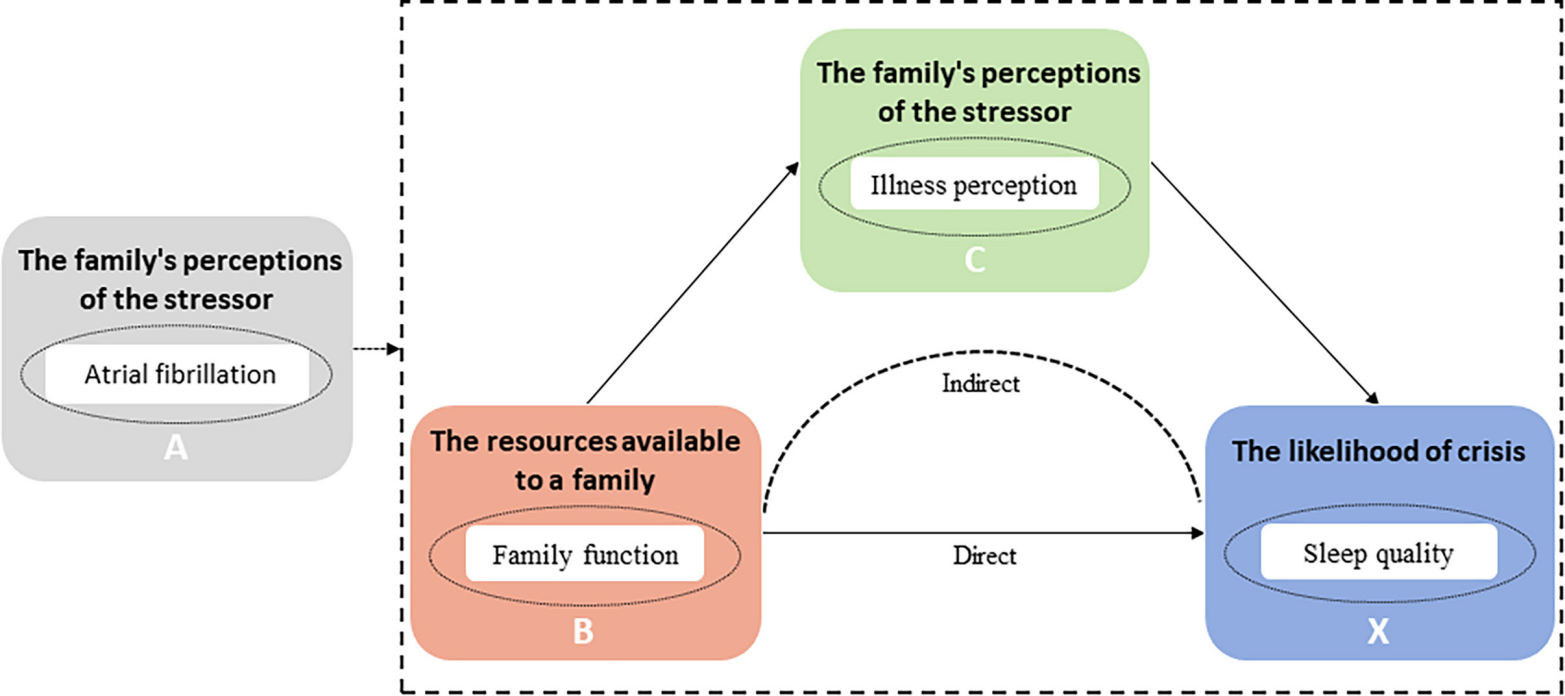
Theoretical model of this study.

The aim of this study was to verify the mediating effect of illness perception on the relationship between family function and sleep quality in patients with AF. To achieve this, we proposed three hypotheses: (1) the sleep quality of patients with AF is poor; (2) there is a positive correlation between perceived family function and sleep quality in patients with AF; and (3) the relationship between perceived family function and sleep quality in patients with AF is mediated by illness perception. The theoretical model illustrating these relationships is presented in Figure 1.

### 3.2 Research Design

A descriptive, cross‐sectional survey design was used for this study.

### 3.3 Settings

The study was conducted between November 2020 and November 2021 in the inpatient ward of a third‐grade Class A hospital located in Wuhan, China. This setting allowed for the recruitment of patients who were actively being treated for AF and thus could provide insights into the real‐world experiences and perceptions of this patient population.

### 3.4 Population and Sample

The target population consisted of hospitalised patients diagnosed with AF according to body surface electrocardiogram or 24‐h dynamic electrocardiogram. Inclusion criteria were as follows: (1) patients with AF diagnosis, (2) age ≥ 18 years, (3) ability to communicate and (4) informed consent for voluntary participation. Patients were excluded if they had complications of important organs (brain, liver and kidney), a history of mental illness or cognitive dysfunction, malignant tumours or other serious cardiovascular diseases.

To ensure the reliability of the study findings, the sample size was calculated using the N:q ratio, that is, the sample size‐to‐parameter ratio ranges from 10 to 20 (Jackson 2003; Ni et al. 2023). Adjusting for a potential nonresponse rate of 10%, the target sample size was set at 200. Convenience sampling was used to recruit 200 patients with AF, and after excluding responses with missing data or obvious errors, a final sample of 191 valid responses was obtained for analysis.

### 3.5 Instruments and Variables

#### 3.5.1 Social‐Demographic and Clinical Characteristics

Sociodemographic characteristics including sex, age, marital status, education level, living with partner, employment status, medical insurance type, and monthly income were collected through a structured questionnaire administered to the patients during face‐to‐face interviews. Additionally, clinical characteristics related to AF, such as the duration of AF, type of AF, and severity of AF symptoms assessed by the EHRA (European Heart Rhythm Association) score (Kirchhof et al. 2007) and whether the patients were complicated with other chronic diseases, were extracted from the electronic records of clinical assessments performed by healthcare professionals. Among them, the EHRA score (Kirchhof et al. 2007), a validated tool, classifies AF symptom severity into four categories, ranging from EHRA I (asymptomatic) to EHRA IV (symptoms limiting daily activities).

#### 3.5.2 Sleep Quality

Pittsburgh Sleep Quality Index (PSQI), compiled by Buysse et al. (1989) in 1989, is mainly used to assess the overall sleep performance of respondents in the most recent month. PSQI was composed of 19 self‐report items and 5 other evaluation items, of which only the first 18 self‐report items participated in the score and divided into 7 dimensions: subjective sleep quality, sleep latency, sleep duration, sleep efficiency, sleep disturbance, daytime dysfunction and frequency of sleep medications, with 0 (no difficulty) to 3 (severe difficulty). The sum of scores ranged from 0 (better sleep) to 21 (worst sleep). The total scores of PSQI > 7 indicate poor sleep quality, and the Chinese version of PSQI has been reported to exhibit satisfactory reliability measures, thereby validating its use in research contexts (Liu et al. 1996).

#### 3.5.3 Family Function

The Family APGAR index (APGAR) was developed by Smilkstein (1978) in 1978 to assess patient satisfaction with family function. The index consists of five items grouped into five components: adaptation, partnership, growth, affection and resolve with 0 (hardly ever) to 2 (almost always). The range of the total scores is 0–10, and the higher the scores, the better the family function. A total score of APGAR < 3 indicates severe family dysfunction, 4–6 indicates moderate family dysfunction and 7–10 indicates good family function. In this study, moderate and severe family dysfunction were combined into low family function. It was reported that the test–retest reliability of the index in the Chinese population was 0.73–0.94 (Lv et al. 1999; Nan et al. 2013).

#### 3.5.4 Illness Perception

Brief Illness Perception Questionnaire (BIPQ), simplified by Broadbent et al. (2006) in 2006, is used to evaluate the impact of patients' perceived illness on their lives, their attention to the illness and their emotional perception. There were eight scoring items and one open question in the questionnaire. The eight items include cognitive representation dimension (five items), emotional representation dimension (two items) and illness understanding dimension (one item), with 0 (none) to 10 (seriously affected life). The total scores of the questionnaire ranged from 0 to 80, and the higher the scores represent more negative illness perception. Cronbach's *α* coefficient of the Chinese version of the BIPQ scale was 0.77 (Mei et al. 2015).

### 3.6 Research Validity, Reliability and Rigour

All the questionnaires used in the study had good reliability and validity. All data were collected and processed by the researchers to ensure authenticity and validity of the study. The guideline for Strengthening the Reporting of Observational Studies in Epidemiology (STROBE) was executed and reported, as shown in Appendix S1.

### 3.7 Statistical Analysis

IBM SPSS version 21.0 was used to analyse data. General characteristics and variables of the participants were analysed using descriptive statistics. Differences in sleep quality according to general characteristics were analysed by using independent‐sample *t*‐test and one‐way ANOVA. Multiple linear regression analysis was used to identify independent factors associated with sleep quality. At the same time, we further used Pearson correlation analysis to analyse the correlation among sleep quality, family function and illness perception. Finally, we used SPSS Process macro, version 3.1 (Model 4), to explore the mediating role of illness perception in the relationship between family function and sleep quality (Igartua and Hayes 2021). According to guideline (Hayes 2013), we selected the bias‐corrected (BC) bootstrap method with 5000 samples to test the degree of mediating effect, thus revealing the importance of total effect in path analysis. In addition, to avoid potential confounding bias, in the test of intermediary effect, we control the significant sociodemographic variables in linear regression and the variables that have an empirical relationship with our focus variables in previous studies. All statistical significance was set at *p* ≤ 0.05.

### 3.8 Ethical Considerations

This study received ethical approval from the Ethics Committee of Wuhan University (approval number: 2021YF0049), ensuring adherence to institutional and national research guidelines and the 1964 Helsinki Declaration with subsequent amendments. In implementing the study, we rigorously followed ethical principles. Prior to participation, all eligible individuals were provided with a comprehensive informed consent form outlining the study objectives, procedures, potential risks, benefits and confidentiality measures. Participants were given ample time to review and clarify any uncertainties, and their written informed consent was obtained voluntarily before any data collection commenced.

## 4 Results

### 4.1 Sleep Quality Scores by Participant's Characteristics

The mean age of the participants was 64.13 years (SD = 10.80), and more than half of them (61.8%) were male. Of these, 92.1% were married, 74.3% were inactive or retired and 80.7% had general residents' medical insurance. Sleep quality scores ranged from 1 to 20, with a mean score of 10.57 and a standard deviation of 4.54, with about 71.2% scoring less than 7. In addition, there were differences in sleep quality among participants of different sexes, ages, marital statuses, education levels, employment statuses, medical insurance types, monthly incomes, EHRA scores and whether they had hyperglycaemia (see Table 1).

**TABLE 1:** Sleep quality scores by participant's characteristics (*N* = 191).

Variable	*N* (%)/*M* ± SD	PSQI		
		*M* ± SD	*t*/*F/r*	*p*
Sex			10.563^a^	0.001
Male	118 (61.8%)	9.75 ± 4.32		
Female	73 (38.2%)	11.89 ± 4.61		
Age (years)		—	0.212^c^	0.003
Age range 33–88 years	64.13 ± 10.80			
Marital status			5.323^a^	0.022
Married	176 (92.1%)	10.57 ± 4.47		
Divorced/widowed	15 (7.9%)	13.13 ± 4.68		
Education level			5.181^b^	0.002
Primary school and below	43 (22.5%)	12.14 ± 4.66		
Junior high school	35 (18.3%)	11.54 ± 4.35		
Senior school	50 (26.2%)	10.54 ± 4.55		
College and above	63 (33.0%)	8.97 ± 4.11		
Living with partner			0.946^a^	0.332
Yes	174 (91.1%)	10.47 ± 4.59		
No	17 (8.9%)	11.59 ± 4.00		
Employment status			7.477^a^	0.007
Employed	49 (25.7%)	9.06 ± 4.49		
Inactive or retired	142 (74.3%)	11.08 ± 4.45		
Medical insurance type			4.008^b^	0.009
Public financed medical insurance	30 (15.7%)	9.13 ± 3.88		
General residents' medical insurance	154 (80.7%)	10.91 ± 4.53		
Commercial medical insurance	2 (1.0%)	2.00 ± 1.41		
Self‐financed	5 (2.6%)	12.00 ± 5.24		
Digestive system diseases			3.203^a^	0.075
Yes	35 (18.3%)	11.80 ± 4.11		
No	156 (81.7%)	10.29 ± 4.60		
Monthly income (Renminbi)			6.913^b^	< 0.001
< 1000	12 (6.3%)	8.92 ± 4.96		
1000–2999	55 (28.8%)	12.62 ± 4.24		
3000–5000	65 (34.0%)	10.94 ± 4.41		
> 5000	59 (30.9%)	8.59 ± 4.02		
Hypertension			0.763^a^	0.384
Yes	93 (48.7%)	10.86 ± 4.58		
No	98 (51.3%)	10.29 ± 4.51		
Hyperglycaemia			5.685^a^	0.018
Yes	25 (13.1%)	12.56 ± 4.06		
No	166 (86.9%)	10.27 ± 4.55		
Dyslipidaemia			0.865^a^	0.354
Yes	37 (19.4%)	11.19 ± 4.13		
No	154 (80.6%)	10.42 ± 4.63		
AF duration			0.664^b^	0.575
< 1 years	43 (22.5%)	10.26 ± 4.65		
1–4 years	84 (44.0%)	10.40 ± 4.46		
5–9 years	32 (16.8%)	11.59 ± 4.94		
≥ 10 years	32 (16.7%)	10.38 ± 4.25		
Types of AF			0.566^b^	0.569
Paroxysmal AF	146 (76.4%)	10.68 ± 4.54		
Persistent AF	38 (19.9%)	9.95 ± 4.42		
Permanent AF	7 (3.7%)	11.57 ± 5.53		
Severity of AF symptoms (EHRA score)			9.527^b^	< 0.001
EHRA I	28 (14.7%)	9.32 ± 3.95		
EHRA II	46 (24.1%)	8.43 ± 3.76		
EHRA III	82 (42.9%)	11.05 ± 4.72		
EHRA IV	35 (18.3%)	13.23 ± 3.97		

Abbreviations: M, Mean; SD, Standard deviation.

^a^ *t*: Independent‐Samples *t*‐tests.

^b^ *F*: one‐way ANOVA.

^c^ *r*: Pearson Correlation analysis.

### 4.2 Multiple Linear Regression Analysis

Previous studies have found that there are significant differences in sleep quality among patients with AF of different sexes (Risom et al. 2018; Szymanski et al. 2014), ages (Szymanski et al. 2014), blood pressure (Szymanski et al. 2014), living with partner status (Risom et al. 2018) and severity of AF symptoms (Risom et al. 2018; Szymanski et al. 2014). Therefore, to adjust more confounders, in addition to the variables with *p*‐value < 0.05 in univariate analysis, we inputted additional variables (blood pressure and living with partner) that were associated with sleep quality in patients with AF into the multivariate linear regression model, although they did not show any significant differences in univariate analysis. The assignment of independent variables is shown in Table 2. The results showed that the factors affecting the sleep quality of patients with AF included medical insurance type (*B* = −9.474, 95% CI [−16.373, −2.622]) and EHRA score (*B* = 2.134, 95% CI [0.609, 3.659]; *B* = 3.277, 95% CI [1.365, 5.189]); please see Table 3. These factors accounted for 21.5% of the total variation of the regression equation in sleep quality in patients with AF. In the process of analysis, the problem of multicollinearity was not found, and the range of tolerance was 0.142–0.884, all greater than 0.1. Also, the variance inflation factor was between 1.131 and 7.240 and did not exceed the standard point of 10.

**TABLE 2:** Table of independent variable assignment.

Independent variable	Assignment (Dummy coded)
Sex	Male = 0; Female = 1
Age	Bring in the original scores
Marital status	Divorced/Widowed = 0; Married = 1
Living with partner	No = 0; Yes = 1
Educational level	Primary school and below (Z1 = 0, Z2 = 0, Z3 = 0); Junior high school (Z1 = 1, Z2 = 0, Z3 = 0); Senior school (Z1 = 0, Z2 = 1, Z3 = 0); College and above (Z1 = 0, Z2 = 0, Z3 = 1)
Employment status	Inactive or retired = 0; Employed = 1
Medical insurance type	Self‐financed (Z1 = 0, Z2 = 0, Z3 = 0); Public financed medical insurance (Z1 = 1, Z2 = 0, Z3 = 0); General residents' medical insurance (Z1 = 0, Z2 = 1, Z3 = 0); Commercial medical insurance (Z1 = 0, Z2 = 0, Z3 = 1)
Monthly income (RMB)	< 1000 (Z1 = 0, Z2 = 0, Z3 = 0); 1000–2999 (Z1 = 1, Z2 = 0, Z3 = 0); 3000–4999 (Z1 = 0, Z2 = 1, Z3 = 0); ≥ 5000 (Z1 = 0, Z2 = 0, Z3 = 1)
Hypertension	Yes = 0; No = 1
Hyperglycaemia	Yes = 0; No = 1
Severity of AF symptoms	EHRA II (Z1 = 0, Z2 = 0, Z3 = 0); EHRA I (Z1 = 1, Z2 = 0, Z3 = 0); EHRA III (Z1 = 0, Z2 = 1, Z3 = 0); EHRA IV (Z1 = 0, Z2 = 0, Z3 = 1)

**TABLE 3:** Multiple linear regression analysis of the factors influencing sleep quality (PSQI scores) in participants (*N* = 191).

Independent variable	*B*	SE	*β*	*t*	*p*	95%CI
Constant	8.619	4.301		2.004	0.047	[0.129, 17.108]
Sex (vs. Male)	1.356	0.696	0.145	1.949	0.053	[−0.018, 2.729]
Age	0.034	0.040	0.080	0.851	0.396	[−0.045, 0.112]
Marital status (vs. Divorced/Widowed)	−1.065	1.437	−0.063	−0.741	0.460	[−3.900, 1.771]
Living with partner (vs. No)	0.449	1.248	0.028	0.360	0.719	[−2.015, 2.913]
Educational level (vs. Primary school and below)						
Junior high school	1.690	1.056	0.144	1.599	0.112	[−0.396, 3.775]
Senior school	0.724	1.030	0.070	0.703	0.483	[−1.308, 2.757]
College and above	0.169	1.115	0.018	0.152	0.880	[−2.033, 2.371]
Employment status (vs. Employed)	0.155	0.969	0.015	0.160	0.873	[−1.757, 2.067]
Medical insurance type (vs. Self‐financed)						
Public financed medical insurance	−2.475	2.123	−0.199	−1.166	0.245	[−6.666, 1.717]
General residents' medical insurance	−1.607	1.983	−0.140	−0.811	0.419	[−5.521, 2.306]
Commercial medical insurance	−9.497	3.483	−0.213	−2.727	0.007	[−16.373, −2.622]
Monthly income (RMB) (vs. < 1000)						
1000–2999	1.741	1.442	0.174	1.207	0.229	[−1.106, 4.588]
3000–4999	1.017	1.395	0.106	0.729	0.467	[−1.737, 3.771]
≥ 5000	−0.384	1.490	−0.039	−0.258	0.797	[−3.324, 2.557]
Hypertension (vs. Yes)	0.157	0.634	0.017	0.248	0.805	[−1.095, 1.409]
Hyperglycaemia (vs. Yes)	−1.627	0.918	−0.121	−1.772	0.078	[−3.440, 0.186]
Severity of AF symptoms (vs. EHRA II)						
EHRA I	0.776	0.993	0.061	0.782	0.436	[−1.183, 2.735]
EHRA III	2.134	0.773	0.233	2.763	0.006	[0.609, 3.659]
EHRA IV	3.277	0.969	0.280	3.383	0.001	[1.365, 5.189]

*Note:* Model *F* = 3.735, *p* < 0.001; *R* ^2^ = 0.293, Adjusted *R* ^2^ = 0.215.

### 4.3 Descriptive Statistics and Preliminary Correlation Analysis

There was a highly significant relationship among APGAR (*r* = −0.439, *p* < 0.001), BIPQ (*r* = 0.411, *p* < 0.001) and PSQI. In patients with AF, the larger the APGAR or the smaller the BIPQ, the lower the PSQI scores; please see Table 4.

**TABLE 4:** Means, standard deviations and correlations among the variables (*N* = 191).

Variables	*M* ± *SD*	1	2	3
APGAR	7.27 ± 2.38	1		
BIPQ	43.15 ± 11.71	−0.327***	1	
PSQI	10.57 ± 4.54	−0.439***	0.411***	1

*** *p* < 0.001.

### 4.4 Mediation Analyses

**FIGURE 2:**
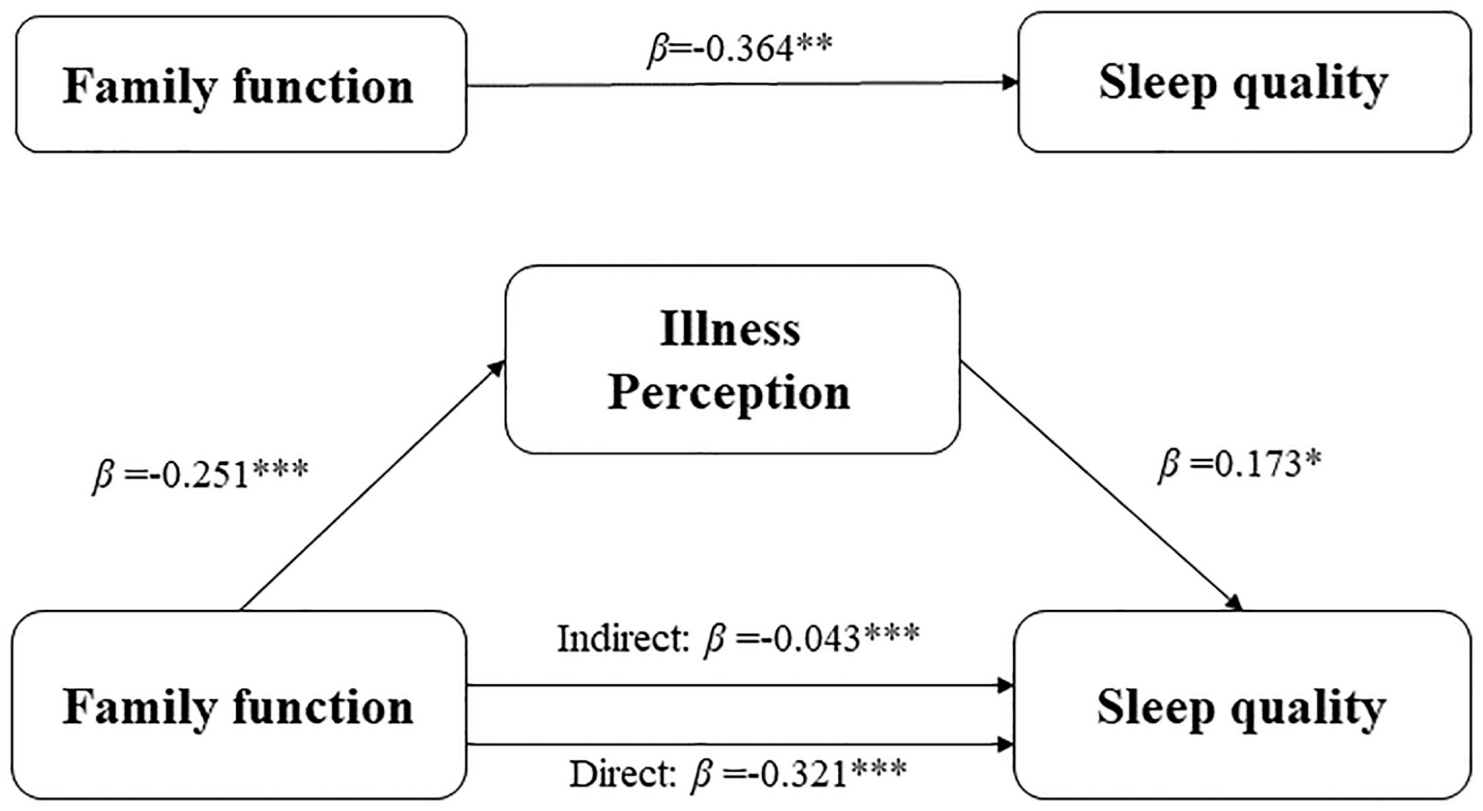
A model diagram of illness perception mediated family function and sleep quality. *p < 0.05, **p < 0.01, ***p < 0.001. The measurement tools for illness perception, family function and sleep quality were BIPQ, APGAR and PSQI, respectively.

Given the experience of previous studies and the results of the multiple linear regression analyses in this study, sex, age, living with partner, medical insurance type, hypertension and EHRA score were set as covariates in this study. The results of the mediation analyses are shown in Tables 5 and 6. Specifically, the APGAR scores were negatively associated with both the BIPQ scores (*β* = −0.251, *p* < 0.001) and PSQI scores (*β* = −0.364, Bootstrap BC 95%CI [−0.933, −0.456]). This indicates that lower family function is associated with poor sleep quality (higher PSQI scores). The indirect effect of family function on sleep quality through illness perception was also significant (*β* = −0.043, Bootstrap BC 95%CI [−0.169, −0.014]), accounting for 11.8% of the total effect. The structural relationships among illness perception, family function and sleep quality are shown in Figure 2.

**TABLE 5:** Mediated analysis model testing (*N* = 191).

Variable	Model 1: Sleep quality (PSQI score)			Model 2: Illness perception (BIPQ score)			Model 3: Sleep quality (PSQI score)		
	*β*	*t*	*p*	*β*	*t*	*p*	*β*	*t*	*p*
Family function (APGAR score)	−0.364	−5.754	< 0.001	−0.251	−3.967	< 0.001	−0.321	−4.922	< 0.001
Illness perception (BIPQ score)							0.173	2.367	0.019
*R*	0.581			0.579			0.597		
*R* ^2^	0.337			0.335			0.357		
*F*	11.567			11.482			11.165		
*p*	< 0.001			< 0.001			< 0.001		

*Note:* Adjusted sex, medical insurance type and EHRA score.

**TABLE 6:** Testing the mediation effect of illness perception (*N* = 191).

Path	Effect	*β*	Boot SE	Bootstrap BC (95%CI)	
				Lower	Upper
Family function (APGAR score) → Illness perception (BIPQ score) → Sleep quality (PSQI score)	Total effect, c	−0.364	0.121	−0.933	−0.456
	Indirect effect, a*b	−0.043	0.040	−0.169	−0.014
	Direct effect, c′	−0.321	0.124	−0.857	−0.366
	Ratio of indirect to total effect mediated (a*b/c)	11.8%			

*Note:* Adjusted sex, medical insurance type and EHRA score.

## 5 Discussion

### 5.1 Summary of Main Results

In this study, our research hypothesis has been verified. Patients with AF reported a mean PSQI scores of 10.57 ± 4.54, and approximately 71.2% reported poor sleep quality, suggesting that sleep quality impairment is very common in patients with AF, similar to the results of previous studies (Risom et al. 2018; Szymanski et al. 2014; Wood, Higgins, and Barnes 2023). In addition, they reported high levels of family function (7.27 ± 2.38) and moderate levels of perceived threat of illness (43.15 ± 11.71). Among the patients with AF, the better family function (*r* = −0.439, *p* < 0.001), the lower the perceived threat of illness (*r* = 0.411, *p* < 0.001) and the better their sleep quality. In multivariate analysis, type of medical insurance and EHRA score were the main factors associated with sleep quality, with patients with commercial medical insurance (*B* = −9.497, 95% CI [−16.373, −2.622]) maintaining better sleep quality relative to self‐financed patients. Patients with AF symptom severity ratings of EHRA III (*B* = 2.134, 95% CI [0.609, 3.659]) and EHRA IV (*B* = 3.277, 95% CI [1.365, 5.189]) had worse sleep quality relative to those rated EHRA II. The results of the mediation analysis indicated that illness perception played a mediating role in the relationship between family function and sleep quality in patients with AF (*β* = −0.043, Bootstrap BC 95% CI [−0.169, −0.014]).

### 5.2 Factors Related to Sleep Quality

#### 5.2.1 Medical Insurance Type

Our study found that patients with commercial medical insurance reported lower PISQ, that is, better sleep quality relative to self‐financed AF patients. We hypothesised that this relationship may be related to the fact that commercial medical insurance can more greatly diminish the impact of family financial stress on health. A Chinese analysis of hospitalisation costs for 1235 patients with AF in a large general hospital from 2011 to 2019 found that the average hospitalisation cost for patients with AF was Renminbi (RMB) 20,325.1 (37.6% out of pocket) from 2011 to 2017, and the average amount of hospitalisation out‐of‐pocket costs for patients with paroxysmal AF and patients with persistent AF was higher than RMB 30,000 in 2018–2019 (Chen et al. 2021). The individual out‐of‐pocket costs of patients with AF are on the rise, and even under publicly financed medical insurance still face financial risks due to high out‐of‐pocket costs. Commercial medical insurance, as a complementary form of health insurance (Fu 2021), can help patients manage their health to a certain extent at a lower cost of health care services and can bring additional health gains in terms of promoting the health of the population (Chen et al. 2022; Yi, Wei, and Long 2023). As a result, out‐of‐pocket patients with AF are more likely to be plagued by medical economic expenses, resulting in a higher risk of poor sleep quality (Li et al. 2020). The results emphasise the need for ongoing attention to the differences in sleep quality demonstrated by differently insured AF populations and effective preventive measures to minimise patients' concerns about the cost of treating their disease.

#### 5.2.2 Severity of AF Symptoms

The relationship between the severity of AF symptoms and sleep quality appears to be different from previous studies. Both Szymanski et al. (2014) and Risom et al. (2018) found that as the EHRA score level increased, the sleep quality of patients with AF was poorer. In our study, we found that patients with AF scoring EHRA III and EHRA IV both had poorer sleep quality relative to patients scoring EHRA II, but patients scoring EHRA I did not show significant improvement in sleep quality. One possible reason for this is that emotional distress caused by AF itself also significantly affects the overall well‐being of patients with asymptomatic AF (Savelieva et al. 2001). In addition, because of the concealment of symptoms and the risk of adverse disease outcomes, the management of patients with asymptomatic AF is often more challenging (Boriani et al. 2015). On the one hand, it may increase the level of perceived threat of the disease in patients with asymptomatic AF, and on the other hand, the level of perceived risk of the disease prognosis in patients is not the same as that received by them, it may not be as important as it is in the case of patients with AF who are not at risk. Furthermore, in terms of management strategies, Boriani et al. (2015) found that rhythm control and medications were applied less frequently in patients with asymptomatic AF relative to those with symptomatic AF. Therefore, the imbalance between the patient's perceived level of prognostic risk and the health prescriptions they receive may make them more hypersensitive to the disease process and affect sleep quality. In summary, we suggest that the recognition of sleep patterns in asymptomatic AF patients should not be reduced daily, but on the contrary, sleep quality care should be enhanced in the same way as in symptomatic AF patients.

### 5.3 Relationship Among Family Function, Illness Perception and Sleep Quality

APGAR scores in AF patients had a significant negative predictive effect on PSQI scores, which is similar to the results of previous studies, that is, obtaining a high level of family support is more conducive to promoting an individual's healthy sleep quality (Ailshire and Burgard 2012; Mousavi et al. 2022; Stafford et al. 2017), which also supports our Hypothesis 2. It is not difficult to understand that the emotional comfort provided by an intimate and warm family environment is more likely to help individuals reduce their alertness to the environment and stress, and to promote the evaluation of healthy sleep (Dahl and El‐Sheikh 2007). On the contrary, tension and harsh family relationships, both affect the level of social support that individuals receive from their families and as a source of stress which may exacerbate the problems experienced by the patient. This stress from the family has been found to be more powerful than the predictive effect of family support on sleep problems (Ailshire and Burgard 2012). Pietromonaco and Collins (2017) also reported that the close relationship between the patient and family can buffer the individual from the negative effects of stress, attenuate the individual's stress assessment and cardiovascular reactivity and promote recovery and resilience (Pietromonaco and Collins 2017). Therefore, our study suggests that exploring a family‐centred nursing intervention model may be helpful in alleviating sleep disorders in patients with AF.

In addition, Hypothesis 3 was supported because a mediating role of illness perception was detected in the relationship between family function and sleep quality. This implies that family function can improve sleep quality by improving illness perception. Several explanations can be proposed for this relationship. First, good family function may enhance mutual support and understanding among family members, which may help patients with AF to improve their understanding and perception of their health status, that is, to generate a positive illness perception (Ding et al. 2023). In addition, distorted illness perception may lead to a greater psychological burden of anxiety, depression and other diseases in patients with AF (McCabe and Barnason 2012), and impaired mental health may interfere with patients' sleep patterns (Risom et al. 2018). Moreover, we cannot ignore the link between illness perception and symptoms of AF, which helps to explain the predictive role of illness perception on sleep quality. AF symptoms stem from patients' cognitive interpretations of bodily sensations, which can affect the sleep process because of the discomfort it brings (Wood, Higgins, and Barnes 2023). Thus, patients with AF with positive illness perception are more likely to benefit from the support of good family function and cultivate mindfulness to cope with the stresses and challenges of the disease process, which can help manage the overall disease experience.

### 5.4 Implications of the Study

The findings of this study have important implications for nursing practice. By demonstrating that illness perception plays a significant mediating role in the relationship between family function and sleep quality among patients with AF, nurses are reminded of the crucial need to address not only the physical aspects of the disease but also the emotional and psychological factors that contribute to patients' overall well‐being. Improving family function and fostering a positive illness perception can directly enhance sleep quality, a known factor influencing the course of AF. Nurses should engage in interventions that strengthen family support systems and educate patients on the importance of adopting a positive illness mindset. Such efforts can potentially lead to better sleep outcomes and ultimately contribute to improved health outcomes for patients with AF. Furthermore, our results highlight the significance of considering medical insurance type and AF symptom severity in managing sleep quality, emphasising the need for tailored nursing interventions that consider patients' socioeconomic and clinical characteristics.

### 5.5 Limitations

This study is one of the first to explore how illness perception plays a role in the relationship between family function and sleep quality in patients with AF. However, some limitations remain; first, although we analysed the associations between variables based on theoretical models, the cross‐sectional design precluded inferences of causality, and further longitudinal and intervention studies should be conducted in the future to extend our understanding of the causal relationships among family function, illness perception and sleep quality. Second, the study sample was drawn from only one hospital in Hubei Province, China, and it was not possible to determine whether the respondents were representative of AF patients in all regions of China or other countries. Finally, all questionnaires were self‐reported, which may not reflect the actual situation of patients.

## 6 Conclusions

Our main findings demonstrated that the sleep quality of patients with AF is poor, and the type of medical insurance and EHRA score are independent indicators related to their sleep quality. Improving family function and helping patients cultivate positive illness cognition can directly and indirectly improve the sleep quality of patients with AF, which may be a promising way to improve the sleep quality of patients with AF. Therefore, nurses should focus on the family function of patients with AF, motivate family members to provide emotional support and better communication for patients with AF, optimise patients' views on the disease and take these measures as part of the overall care plan for disease management, ultimately protecting their sleep quality.

## Author Contributions

L.X.Q. and A.Z.F. conceived the original idea, contributed to the study design and completed the study analysis. A.Z.F. interpreted the results and wrote the original draft. X.Y.Y., Z.X. and F.P. commented on previous versions of the manuscript. L.Y.P., C.Z.X. and Y.L.P. supervised and reviewed the entire process. All authors have read and approved the final manuscript.

## Conflicts of Interest

The authors declare no conflicts of interest.

## Supporting information


Appendix S1.

## Data Availability

The data related to the findings of this study are available from the first author (Xiaoqing Lu) upon reasonable request.

## References

[nop270085-bib-0001] Ailshire, J. A. , and S. A. Burgard . 2012. “Family Relationships and Troubled Sleep Among U.S. Adults: Examining the Influences of Contact Frequency and Relationship Quality.” Journal of Health and Social Behavior 53, no. 2: 248–262. 10.1177/0022146512446642. 22653715 PMC3674886

[nop270085-bib-0002] Boriani, G. , C. Laroche , I. Diemberger , et al. 2015. “Asymptomatic Atrial Fibrillation: Clinical Correlates, Management, and Outcomes in the EORP‐AF Pilot General Registry.” American Journal of Medicine 128, no. 5: 509–518.e2. 10.1016/j.amjmed.2014.11.026. 25534423

[nop270085-bib-0003] Broadbent, E. , K. J. Petrie , J. Main , and J. Weinman . 2006. “The Brief Illness Perception Questionnaire.” Journal of Psychosomatic Research 60, no. 6: 631–637. 10.1016/j.jpsychores.2005.10.020. 16731240

[nop270085-bib-0004] Buysse, D. J. , C. F. Reynolds , T. H. Monk , S. R. Berman , and D. J. Kupfer . 1989. “The Pittsburgh Sleep Quality Index: A New Instrument for Psychiatric Practice and Research.” Psychiatry Research 28, no. 2: 193–213. 10.1016/0165-1781(89)90047-4. 2748771

[nop270085-bib-0005] Chen, X. , D. Guo , H. Tan , et al. 2022. “Can Supplementary Private Health Insurance Further Supplement Health.” Frontiers in Public Health 10: 961019. 10.3389/fpubh.2022.961019. 36238234 PMC9552012

[nop270085-bib-0006] Chen, Y. , H. Cui , G. Yonghong , and L. Sun . 2021. “Analysis and Management Suggestions on Hospitalization Expenses of Atrial Fibrillation in a Large General Hospital.” China Health Insurance 04: 59–62. 10.19546/j.issn.1674-3830.2021.4.014.

[nop270085-bib-0007] Christensen, M. A. , S. Dixit , T. A. Dewland , et al. 2018. “Sleep Characteristics That Predict Atrial Fibrillation.” Heart Rhythm 15, no. 9: 1289–1295. 10.1016/j.hrthm.2018.05.008. 29958805 PMC6448388

[nop270085-bib-0008] Chung, M. K. , L. L. Eckhardt , L. Y. Chen , et al. 2020. “Lifestyle and Risk Factor Modification for Reduction of Atrial Fibrillation: A Scientific Statement From the American Heart Association.” Circulation 141, no. 16: e750–e772. 10.1161/CIR.0000000000000748. 32148086

[nop270085-bib-0009] Dahl, R. E. , and M. El‐Sheikh . 2007. “Considering Sleep in a Family Context: Introduction to the Special Issue.” Journal of Family Psychology 21, no. 1: 1–3. 10.1037/0893-3200.21.1.1. 17371104

[nop270085-bib-0010] Deschênes, S. S. , R. J. Burns , E. Graham , and N. Schmitz . 2019. “Depressive Symptoms and Sleep Problems as Risk Factors for Heart Disease: A Prospective Community Study.” Epidemiology and Psychiatric Sciences 29: e50. 10.1017/S2045796019000441. 31426879 PMC8061258

[nop270085-bib-0011] Ding, Y.‐M. , C.‐P. Liu , H.‐X. Xu , et al. 2023. “Effect of Social Support on Illness Perception in Patients With Atrial Fibrillation During “Blanking Period”: Mediating Role of Sense of Mastery.” Nursing Open 10, no. 1: 115–122. 10.1002/nop2.1284. 35855521 PMC9748061

[nop270085-bib-0012] Fu, X. 2021. “Financial Protection Effects of Private Health Insurance: Experimental Evidence From Chinese Households With Resident Basic Medical Insurance.” International Journal for Equity in Health 20: 122. 10.1186/s12939-021-01468-5. 34001149 PMC8130397

[nop270085-bib-0013] Gable, S. L. , and A. Bedrov . 2022. “Social Isolation and Social Support in Good Times and Bad Times.” Current Opinion in Psychology 44: 89–93. 10.1016/j.copsyc.2021.08.027. 34600413

[nop270085-bib-0014] Hayes, A. F. 2013. Introduction to Mediation, Moderation, and Conditional Process Analysis: A Regression‐Based Approach. New York, NY: Guilford Press.

[nop270085-bib-0015] Hill, R. 1949. Families Under Stress: Adjustment to the Crises of War Separation and Return, x–443. New York, NY: Harper.

[nop270085-bib-0016] Igartua, J. J. , and A. F. Hayes . 2021. “Mediation, Moderation, and Conditional Process Analysis: Concepts, Computations, and Some Common Confusions.” Spanish Journal of Psychology 24: e49. 10.1017/sjp.2021.46. 35923144

[nop270085-bib-0017] Jackson, D. L. 2003. “Revisiting Sample Size and Number of Parameter Estimates: Some Support for the N : Q Hypothesis.” Structural Equation Modeling‐a Multidisciplinary Journal 10, no. 1: 128–141. 10.1207/s15328007sem1001_6.

[nop270085-bib-0018] Jurczak, C. A. , and R. Hill . 1950. “Families under Stress.” American Catholic Sociological Review 11, no. 1: 45. 10.2307/3706921.

[nop270085-bib-0019] Kirchhof, P. , A. Auricchio , J. Bax , et al. 2007. “Outcome Parameters for Trials in Atrial Fibrillation: Recommendations From a Consensus Conference Organized by the German Atrial Fibrillation Competence NETwork and the European Heart Rhythm Association.” Europace 9, no. 11: 1006–1023. 10.1093/europace/eum191. 17897925

[nop270085-bib-0020] Kwon, Y. , R. J. Koene , A. R. Johnson , G.‐M. Lin , and J. D. Ferguson . 2018. “Sleep, Sleep APNEA and Atrial Fibrillation: Questions and Answers.” Sleep Medicine Reviews 39: 134–142. 10.1016/j.smrv.2017.08.005. 29029984

[nop270085-bib-0021] Leventhal, H. , D. Meyer , and D. Nerenz . 1980. “The Common Sense Representation of Illness Danger.” In Medical Psychology Volume II, edited by S. Rachman , 17–30. New York, NY: Pergamon Press.

[nop270085-bib-0022] Li, N. , G. Xu , G. Chen , and X. Zheng . 2020. “Sleep Quality Among Chinese Elderly People: A Population‐Based Study.” Archives of Gerontology and Geriatrics 87: 103968. 10.1016/j.archger.2019.103968. 31751901

[nop270085-bib-0023] Lippi, G. , F. Sanchis‐Gomar , and G. Cervellin . 2021. “Global Epidemiology of Atrial Fibrillation: An Increasing Epidemic and Public Health Challenge.” International Journal of Stroke 16, no. 2: 217–221. 10.1177/1747493019897870. 31955707

[nop270085-bib-0024] Liu, X. , M. Tang , H. Lei , et al. 1996. “Reliability and Validity of the Pittsburgh Sleep Quality Index.” Chinese Journal of Psychiatry 02: 103–107.

[nop270085-bib-0025] Lv, F. , G. Zeng , S. Liu , T. Zhong , and Z. Zhan . 1999. “A Study on Validity and Reliability of the Family APGAR.” Chinese Journal of Public Health 11: 27–28.

[nop270085-bib-0026] McCabe, P. J. , and S. A. Barnason . 2012. “Illness Perceptions, Coping Strategies, and Symptoms Contribute to Psychological Distress in Patients With Recurrent Symptomatic Atrial Fibrillation.” Journal of Cardiovascular Nursing 27, no. 5: 431–444. 10.1097/JCN.0b013e31821e7ab1. 21743342

[nop270085-bib-0027] Mei, Y. , H. Li , Y. Yang , et al. 2015. “Reliability and Validity of Chinese Version of the Brief Illness Perception Questionnaire in Patients With Breast Cancer.” Journal of Nursing (China) 22, no. 24: 11–14. 10.16460/j.issn1008-9969.2015.24.011.

[nop270085-bib-0028] Morin, D. P. , M. L. Bernard , C. Madias , P. A. Rogers , S. Thihalolipavan , and N. A. M. Estes . 2016. “The State of the Art: Atrial Fibrillation Epidemiology, Prevention, and Treatment.” Mayo Clinic Proceedings 91, no. 12: 1778–1810. 10.1016/j.mayocp.2016.08.022. 27825618

[nop270085-bib-0029] Mousavi, Z. , M.‐L. Tran , J. L. Borelli , A. L. Dent , and K. R. Kuhlman . 2022. “The Moderating Role of Gender in the Association Between Quality of Social Relationships and Sleep.” Journal of Behavioral Medicine 45, no. 3: 378–390. 10.1007/s10865-022-00286-6. 35150370 PMC9160110

[nop270085-bib-0030] Nan, H. , P. H. Lee , M. Y. Ni , B. H. Chan , and T. H. Lam . 2013. “Effects of Depressive Symptoms and Family Satisfaction on Health Related Quality of Life: The Hong Kong FAMILY Study.” PLoS One 8, no. 3: e58436. 10.1371/journal.pone.0058436. 23516480 PMC3597640

[nop270085-bib-0031] Nelson, K. L. , J. E. Davis , and C. F. Corbett . 2022. “Sleep Quality: An Evolutionary Concept Analysis.” Nursing Forum 57, no. 1: 144–151. 10.1111/nuf.12659. 34610163

[nop270085-bib-0032] Ni, Y. X. , D. Wu , Y. Bao , J. P. Li , and G. Y. You . 2023. “The Mediating Role of Psychological Needs on the Relationship Between Perceived Organizational Support and Work Engagement.” International Nursing Review 70, no. 2: 204–210. 10.1111/inr.12797. 35962469

[nop270085-bib-0033] Odutayo, A. , C. X. Wong , A. J. Hsiao , S. Hopewell , D. G. Altman , and C. A. Emdin . 2016. “Atrial Fibrillation and Risks of Cardiovascular Disease, Renal Disease, and Death: Systematic Review and Meta‐Analysis.” BMJ (Clinical Research Ed.) 354: i4482. 10.1136/bmj.i4482. 27599725

[nop270085-bib-0034] Pietromonaco, P. R. , and N. L. Collins . 2017. “Interpersonal Mechanisms Linking Close Relationships to Health.” American Psychologist 72, no. 6: 531–542. 10.1037/amp0000129. 28880100 PMC5598782

[nop270085-bib-0035] Risom, S. S. , P. Fevejle Cromhout , D. Overgaard , J. Hastrup Svendsen , and S. Kikkenborg Berg . 2018. “Effect of Rehabilitation on Sleep Quality After Ablation for Atrial Fibrillation.” Journal of Cardiovascular Nursing 33, no. 3: 261–268. 10.1097/JCN.0000000000000476. 29271795 PMC5908260

[nop270085-bib-0036] Rosino, M. 2016. “ABC‐X Model of Family Stress and Coping.” In Encyclopedia of Family Studies, 1–6. John Wiley & Sons, Ltd. Wuhan University (approval number: 2021YF0049). 10.1002/9781119085621.wbefs313.

[nop270085-bib-0037] Savelieva, I. , M. Paquette , P. Dorian , B. Lüderitz , and A. J. Camm . 2001. “Quality of Life in Patients With Silent Atrial Fibrillation.” Heart 85, no. 2: 216–217. 10.1136/heart.85.2.216. 11156677 PMC1729617

[nop270085-bib-0038] Smilkstein, G. 1978. “The Family APGAR: A Proposal for Family Function Test and Its Use by Physicians.” Journal of Family Practice 6, no. 6: 1231–1239. 660126

[nop270085-bib-0039] Stafford, M. , R. Bendayan , U. Tymoszuk , and D. Kuh . 2017. “Social Support From the Closest Person and Sleep Quality in Later Life: Evidence From a British Birth Cohort Study.” Journal of Psychosomatic Research 98: 1–9. 10.1016/j.jpsychores.2017.04.014. 28554363 PMC5478069

[nop270085-bib-0040] Szymanski, F. M. , K. J. Filipiak , G. Karpinski , A. E. Platek , and G. Opolski . 2014. “Occurrence of Poor Sleep Quality in Atrial Fibrillation Patients According to the EHRA Score.” Acta Cardiologica 69, no. 3: 291–296. 10.1080/ac.69.3.3027832. 25029874

[nop270085-bib-0041] van den Dries, C. J. , S. van Doorn , F. H. Rutten , et al. 2020. “Integrated Management of Atrial Fibrillation in Primary Care: Results of the ALL‐IN Cluster Randomized Trial.” European Heart Journal 41, no. 30: 2836–2844. 10.1093/eurheartj/ehaa055. 32112556 PMC7421774

[nop270085-bib-0042] van Straten, A. , T. van der Zweerde , A. Kleiboer , P. Cuijpers , C. M. Morin , and J. Lancee . 2018. “Cognitive and Behavioral Therapies in the Treatment of Insomnia: A Meta‐Analysis.” Sleep Medicine Reviews 38: 3–16. 10.1016/j.smrv.2017.02.001. 28392168

[nop270085-bib-0043] Wood, K. A. , M. K. Higgins , and A. H. Barnes . 2023. “Self‐Reported Sleep Quality Before and After Atrial Fibrillation Ablation.” Journal of Cardiovascular Nursing 38, no. 2: E78–E86. 10.1097/JCN.0000000000000909. 35389925 PMC9532466

[nop270085-bib-0044] Yi, C. , L. Wei , and C. Long . 2023. “Does Medical Insurance Improve Health? An Empirical Analysis From China.” International Journal of Health Planning and Management 38, no. 3: 829–846. 10.1002/hpm.3628. 36862606

[nop270085-bib-0045] Zhang, Y. 2018. “Family Functioning in the Context of an Adult Family Member With Illness: A Concept Analysis.” Journal of Clinical Nursing 27, no. 15–16: 3205–3224. 10.1111/jocn.14500. 29700875 PMC6105391

[nop270085-bib-0046] Zhou, L. , Y. Wang , S. Li , Y. Hou , X. Zhang , and Y. Wang . 2022. “Death trend and prediction of incidence and mortality of atrial fibrillation in Chinese residents from 1990 to 2019.” Chinese Journal of Prevention and Control of Chronic Diseases 30, no. 06: 410–414. 10.16386/j.cjpccd.issn.1004-6194.2022.06.003.

